# A Virtual Clinic for Diabetes Self-Management: Pilot Study

**DOI:** 10.2196/jmir.1111

**Published:** 2009-03-30

**Authors:** Amy Jennings, John Powell, Natalie Armstrong, Jackie Sturt, Jeremy Dale

**Affiliations:** ^2^Department of Health Sciences (Social Science Research Group)University of LeicesterLeicesterUK; ^1^Health Sciences Research InstituteWarwick Medical SchoolUniversity of WarwickCoventryUK

**Keywords:** Internet, diabetes mellitus, intervention studies, virtual systems, self-efficacy

## Abstract

**Background:**

Internet-based interventions to assist in diabetes management have the potential to provide patients with the information and support they need to become effective self-managers.

**Objective:**

To assess the feasibility, acceptability, and effectiveness of an Internet-based virtual clinic designed to facilitate self-management in patients who used insulin pumps to manage their diabetes.

**Methods:**

For a period of 6 months, 17 patients joined the virtual clinic. The system allowed patients to communicate with health professionals, interact with peers and access information. HbA1c, quality of life, and self-efficacy were monitored at baseline and after 6 months. Questionnaires and qualitative interviews examined patient experiences.

**Results:**

Participants found the virtual clinic easy to use and positively rated its design. Peer support was the most valued aspect and the discussion boards the most used component. All participants highly rated the virtual clinic in terms of improving communication with peers, but few agreed it had improved communication with health care professionals. No significant improvements in physiological and psychological measurements were found. Regarding HbA1c measurements, there was no significant difference found between the pre- and post-test results (P = .53). Mean ADDQoL scores at baseline were -2.1 (SD 1.1, range -3.4 to -0.5) compared to -2.0 (SD 1.2, range, -4.6 to -0.4) post-test (n = 12), (P = .62). Surprisingly, patients’ confidence in their ability to perform self-care tasks was found to be significantly reduced from baseline to follow up (P = .045).

**Conclusions:**

An Internet-based system to aid the management of diabetes appears feasible and well accepted by patients. The pilot study did not identify evidence of an impact on improving quality of life or self-efficacy in patients who used insulin pump therapy.

## Introduction

The Diabetes Control and Complications Trial has conclusively shown that effective control of blood glucose levels delays the onset and slows the progression of diabetes complications [1]. The day-to-day management of diabetes is carried out almost exclusively by the patient and can often be complex and emotionally challenging. To enable patients to be effective self-managers of their diabetes, they need to be provided with the information and support necessary to make informed decisions [2]. Internet-based interventions to aid self-management have the potential to assist patients by offering access to these resources from their own homes, schools, or workplaces and at times when they are most in need of them.

A number of recent Internet-based interventions have been reported on for use with patients with diabetes [3-10], and a pilot study has shown the feasibility of patients with type 2 diabetes co-managing their condition from home [11]. Most of the studies have assessed the usage or usability of their telemedicine systems [3-6,8-11]; some have assessed biological measures, namely HbA1c [3-5,7,9,11]; and one has reported on psychological measurements [5]. Results often indicate improvements in HbA1c values but limited improvements in psychological measurements. Many of the systems have shown feasibility and potential benefits for improvement of diabetes care.

The systems developed for these interventions were often based on the uploading of biological measurements, whilst others provided patients with access to online self-management “coaches”. As a result, these interventions have commonly used complex and bespoke systems; few have used freely available communities. In order for an Internet-based system to be effective, it needs to employ a simple user interface to collect a minimum amount of data [12]. In the current study, a pre-existing virtual clinic prototype was developed as an intervention to aid diabetes self-management, one aimed at patients who used insulin pumps to manage their diabetes.

Continuous subcutaneous insulin infusion (CSII), or pump therapy, is a method of administering insulin over twenty-four hours via a small needle or cannula inserted under the skin. The pump delivers insulin continuously with an additional boost programmed and administered by the patient to match food or reduce raised blood glucose levels [13]. There are a number of reasons why patients may be recommended for CSII. These include inadequate glycemic control with other treatment options, marked variability in glucose on a day-to-day basis, a history of hyperglycemia unawareness, a need for flexibility in lifestyle, pregnancy, insulin sensitivity and low insulin requirements [14]. This particular group has not been studied in any of the previous interventions referenced, despite insulin pump patients showing a great deal of interest in their condition and motivation towards self-management [15].

Community support is believed to be a fundamental aspect of disease self-management, and when peer-support elements are incorporated into Internet-based interventions for diabetes, they are often the most used components [5,10]. The benefits of peer support in relation to health include: decreased feelings of isolation, promotion of positive psychological states and increased motivation, deterring maladaptive behaviors, and providing information on the benefits of behaviors that positively influence health [16]. Studies have shown that discussion forums and chat rooms can have a positive effect on participants by helping them to cope better with diabetes [17].

Self-efficacy can also increase the successful self-management of diabetes. The theory of self-efficacy proposes that an individual’s confidence in their ability to perform a certain behavior influences which behavior they will engage in, how much effort will be expended, and how long they will persist in it [18]. Interventions based on self-efficacy theory have been shown to be significantly more effective than those that are not [19].

In addition to ensuring that the content of an intervention increases self-efficacy, it is important that a system meets patient needs and is designed in conjunction with potential users. Hence, extensive stakeholder consultation [20] and preliminary testing [21] were undertaken to ensure public and patient involvement at all stages of the system’s development. Following this patient-centered approach, we report here on pilot testing of the Internet-based virtual clinic for patients using insulin pumps to manage their diabetes. We aimed to explore the feasibility, acceptability, and effectiveness of the system.

## Methods

### System Design

**Figure 1:**
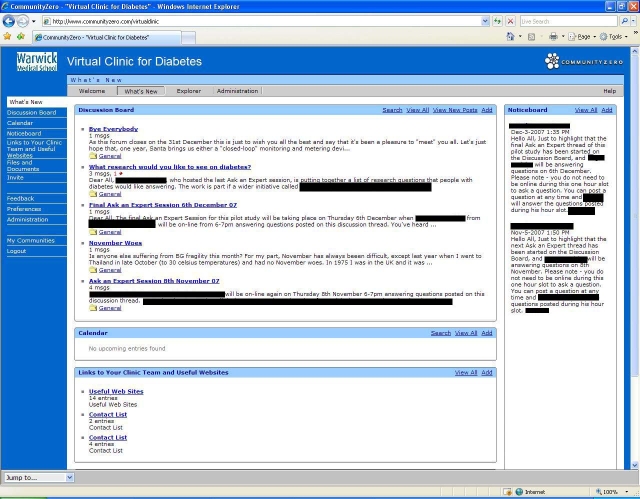
Screenshots of the virtual clinic: homepage

**Figure 2:**
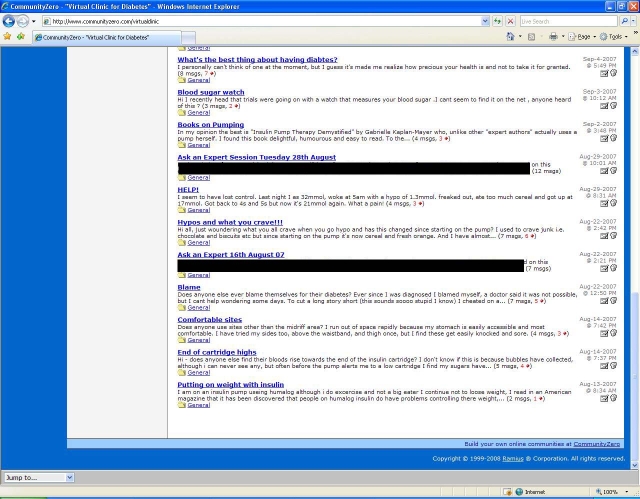
Screenshots of the virtual clinic: discussion forum

The virtual clinic system offered three main Internet-based functions: communication with health professionals, interaction with peers, and access to information (Figure 1 and Figure 2). The site was password protected and only available to the participants involved and those working directly on the study.

Communication with health professionals was provided via 6 online “ask an expert” sessions conducted with diabetes specialists not directly involved with the patients care. These sessions were conducted via the sites asynchronous discussion forums which were open to all who could access the site. Participants were also able to confidentially email their own health professionals at any time and were told to expect a reply within two working days. Interaction with peers was provided via discussion boards and synchronous chat. Discussion board moderation was reactive in that the boards were checked regularly by the study coordinator, and participants were encouraged to report any inappropriate postings. Information on diabetes was provided on the site and via Web links to further sources, including Diabetes UK [22] and sites specific to patients using insulin pumps such as Pumpers UK [23] and Promoting Insulin Pump Therapy (INPUT) [24].

### Sample

Participants were recruited by convenience sampling from three UK hospitals in the West and East Midlands. As the sample was drawn on the basis of opportunity, recruitment was convenient and not time consuming; however, it did increase the risk of bias and the possibility of obtaining a non-representative sample.

Clinicians issued recruitment packs, which consisted of a letter inviting patients to participate and an information sheet outlining the research, to all patients in their diabetes clinics who currently used insulin pumps. Patients were informed that participation was voluntary and refusing participation or withdrawing from the study would not affect their current standard of care. Ethical approval for the study was obtained from the West Midlands Multi-Centre Ethics Committee.

All the participants were over 18 years of age, had used an insulin pump for at least 6 months, could communicate effectively in written and spoken English, had Internet access, and self-reported basic computer literacy. While still receiving normal care, participants were asked to use the virtual clinic for a period of 6 months, logging on at least once a week and using the features within it as often as they wished.

### Measures

The feasibility of the virtual clinic system was determined from recruitment, retention, and usage rates; acceptability was monitored by participant evaluation and informal feedback; and effectiveness was measured by a comparison of pre- and post-test results. Outcome measures were selected following a review of similar studies in this area. As this was a pilot study, it was important to choose measures that would provide feedback in a range of areas, including psychological and physiological. This allowed for a thorough assessment of the intervention and its impact on participants.

Demographic information was recorded using a self-report questionnaire at baseline. Pre-study HbA1c values were obtained, with permission, from patients’ files. Self-efficacy was assessed by the Confidence in Diabetes Self-Care Scale (CIDS) [25] and quality of life by the Audit of Diabetes Dependent Quality of Life (ADDQoL) questionnaire [26]. Participants completed questionnaires by hand and returned them by post.

The CIDS scale is a 21-item self-report questionnaire that covers domains of self-care (eg, insulin administration and blood glucose monitoring). The scale was developed to assess self-efficacy, specifically in adults with type 1 diabetes. Its advantages are that it is a short instrument that has shown high reliability and validity. Respondents are required to score items on a five-point Likert scale from 1 (“No, I am sure I cannot”) to 5 (“Yes, I am sure I can”). A total score is then calculated by summing all items and converting them to a 0-100 scale, with higher scores indicating higher self-efficacy [25].

The ADDQoL questionnaire measures patients’ perceptions of the impact of diabetes on their quality of life with the underlying principle that only personally applicable domains are rated by respondents. The questionnaire consists of 18 life domains (eg, family life, employment, and holidays), and users rate the impact of diabetes on each particular domain and the importance of that domain for their quality of life. A score of +9 is the maximum positive impact of diabetes and -9 the maximum negative impact of diabetes [26]. The advantage of using the ADDQoL questionnaire is that, unlike other quality of life measures that only assess patients’ satisfaction with treatment, a broader range of topics influenced by diabetes, its treatment, and any complications are covered. Furthermore, the questionnaire allows patients to rate only those areas of life that are important to their quality of life.

After using the virtual clinic for a 6-month period, the CIDS and ADDQoL questionnaires were reissued for completion, and patients’ latest HbA1c measurements were taken if a new test had been completed during the study period. Participants’ experiences were assessed at the end of the intervention using questionnaires and qualitative interviews. Five participants completed interviews and were purposively selected by usage, age, and gender to ensure the sample was representative of users. All interviews were audio-recorded and transcribed. Usage statistics were available from the system and recorded as the number of page views.

### Statistical Analysis

Paired sample *t* tests were used to evaluate changes from pre- to post-test. A Wilcoxon Signed rank test was used to examine differences in the CIDS scores as they were not normally distributed. Data were considered statistically significant at *P* < .05. Descriptive statistics were used to analyze the usability questionnaires and the qualitative interviews were analyzed using content analysis.

## Results

### Participants

The age range of the participants who joined the virtual clinic (6 males, 11 females) was 22 to 70 years. On average, respondents had been diagnosed with diabetes for 23.5 years (SD 14.0, range 3 - 58) and had used insulin pumps for 2.8 years (SD 1.6, range 1 - 6). All were described as being of white, British ancestry. Those educated to the undergraduate level or above numbered 8 (47%). The participants all described themselves as regular Internet users. The Internet was used everyday by 11 (65%), and most used it more than once a week. On average, respondents used the Internet for 8.9 hours a week (SD 5.9, range 0.25 - 20) (Table 1).

### Feasibility

Through recruitment, 19 patients were invited to take part in the intervention, and 17 joined the virtual clinic. One participant withdrew during the 6-month trial, and 4 participants failed to complete post-test questionnaires, despite using the virtual clinic for the full 6 months.

**Figure 3:**
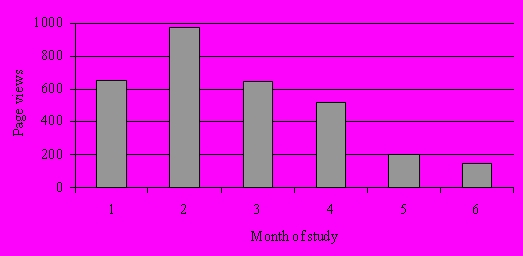
Number of page views per month

Figure 3 shows the usage of the virtual clinic and how this changed over time. In the first month, 648 page views were recorded, and this increased to 971 in the second month. Usage then gradually declined, and only 151 page views were recorded in the final month.

### Acceptability

The system was rated positively by 7 users (58%) for “ease of use” with the remaining 5 (42%) rating it neutrally in the post-test questionnaires. The design of the system was rated positively by 9 users (75%), and only one user gave it a negative rating.

Despite the decline in usage, post-test interviews highlighted the fact that the intervention was very well accepted. Comments from participants included: “[T]he general contact and the facility to be able to get advice, or ask opinions of other people…without it [the virtual clinic] there isn’t that facility” and “[I]t was a very positive experience”. Almost all users agreed that participation in the virtual clinic had reassured them about their diabetes. Only one user expressed on the usability questionnaire that the experience of using the system was “a waste of time”.

It was clear that users found online peer support the most valuable aspect of the intervention. The comfort they took in meeting others of like experience was expressed by two users who said, “just to have communication—to realise you are not the only person in the world like this”;“it has been so nice to realise that your problems are not unique and you’re not on your own trying to solve them”. This probably explains why the discussion board was the most used feature, with participants commenting that involvement was both “useful and reassuring”. There were 34 topics posted on the discussion board and 219 threads. Issues discussed included technical/management problems, seeking and providing emotional support, and general information seeking. No incidences of inappropriate postings were reported.

It became clear that some users posted considerably more threads than others. The most active user posted 25% of the discussion board threads. However, a post-test interview with a very low frequency user revealed that “not being so active didn’t mean that I didn’t think it was useful”, and another user commented that “if there is nothing else you need to know then that is incredibly helpful”. Participation in the discussion board was decribed as useful by 9 users (75%), and all users highly rated the virtual clinic in terms of improving communication with other people with diabetes.

Few users felt that participation in the virtual clinic had improved communication with their clinical team. Although in the follow-up interviews, participants did suggest that some features such as ordering equipment and contacting diabetes nurses were very helpful. Others commented that they did not participate in the “ask-an-expert” sessions because they were uncomfortable with using discussion forums or they simply did not have any questions to ask. In addition, the participants highlighted the fact that that contacting a clinic team for anything other than general questions would be futile, since without providing a detailed background to a problem, professionals would not be able to help.

Participants reported using the links to further information less than the other two components. Many stated they used the links initially but did not revisit them during the intervention. Despite this, only one of the participants said the links were of no use to them.

### Effectiveness

Regarding HbA1c measurements, there was no significant difference found between the pre- and post-test results (*P* = .53). Because measurements were taken from patients’ files, follow up results were only available for participants who had been re-tested by their health care team during the 6-month study period (n = 8, 47%) (Table 1).

Mean ADDQoL scores (Table 1) at baseline were -2.1 (SD 1.1, range -3.4 to -0.5) compared to -2.0 (SD 1.2, range, -4.6 to -0.4) post-test (n = 12), (*P* = .62). Surprisingly, patients’ confidence in their ability to perform self-care tasks was found to be significantly reduced from baseline to follow up (*P* = .045) (Table 1). Mean CIDS scores at baseline were 89.3 (SD 6.64, range 79.8 - 98.8) compared to 83.6 (SD 14.4, range 47.6 - 98.8) post-test (n = 12). However, this finding was due to one outlier in the post-test scores of this small sample (score of 47.6, more than 2.5 standard deviations from the mean). If this outlying case were excluded from the analysis, the CIDS score would not be significantly different post-test (*P* = .08).

**Table 1:** Subject characteristics and test results^a^

ID #	Gender	Age	Years with diabetes	Years on pump	Internet use (hours/week)	HbA1c^b^	HbA1c^c^	CIDS^b^	CIDS^c^	ADDQoL^b^	ADDQoL^c^
1	M	70	36	3	6	7.7%		96.43		-0.11	
2	M	60	12	6	20	8.5%		84.52		-4.06	
3	M	42	3	2	5	9.6%	9.2%	94.05		-3.33	
4	F	38	25	1	15	7.0%	7.0%	94.05	89.29	-0.56	-0.72
5	F	39	16	1	0.25	7.6%		98.81		-0.53	
6	F	32	26	5	4	7.6%	7.2%	91.67	95.24	-1.83	-1.17
7	F	62	58	3	10	6.9%		88.10	84.52	-1.00	-1.75
8	M	59	30	2	14	5.7%		98.81	94.05	-3.44	-1.89
9	M	45	16	3	2	7.9%	8.6%	98.81	94.05	-3.00	-2.78
10	F	52	32	3	4	7.4%	7.2%	79.76	73.81	-2.17	-2.17
11	F	23	6	2	15	5.4%		84.52	47.62	-2.83	-4.61
12	M	47	23	6	10	8.6%	8.6%	91.67	98.81	-0.50	-0.44
13	F	30	22	1	6	8.5%		94.05	95.24	-0.89	-0.67
14	F	22	12	1	10	7.1%		72.62		-1.50	
15	F	59	16	3	14	7.0%		83.33	76.19	-2.56	-2.00
16	F	28	20	3	15	7.3%	8.2%	79.76	77.38	-3.17	-2.78
17	F	67	46	3	1	8.5%	8.8%	86.90	77.38	-3.11	-2.67

^a^Blank spaces indicate data were not available.

^b^Baseline data

^c^Post-test data

## Discussion

### Overview

This study has shown that use of an Internet-based system to facilitate the management of diabetes in insulin pump users is feasible and well accepted by participants. One of the goals of the pilot study was to establish that this intervention could successfully recruit and retain participants. Of the 17 participants in the virtual clinic, 16 used the system throughout the study period, representing a retention rate of 94%.

The number of people using insulin pump therapy to manage their diabetes is growing rapidly. The United Kingdom has seen a dramatic increase with the release of new guidelines in 2003 by the National Institute of Clinical Excellence (NICE) [13]. Individual success with pump therapy requires ongoing education, motivation, and psychological support [14], making insulin pump users important and ideal candidates for interventions of this nature.

The broad age range of the users in our study indicates the system has wide appeal. The fact that most users had been diagnosed with diabetes for over 12 years may have some significance. It is likely that this group of users were well practised and had developed substantial expertise in self-management. Participants were regular Internet users and most were highly educated.

There was a clear decline in use of the intervention over the 6 months. This appears to be a distinct characteristic of Internet-based health interventions and may suggest that the system became less valuable to patients over time. Our usage data follows a typical three-part process of non-usage [27]. There was an initial phase of high usage (months 1 and 2), representing the novelty of the system; a second phase of gradual decline (months 3 and 4), which may indicate that the system does not meet patient expectations or was no longer felt to be so relevant; and a third phase (months 5 and 6) where a stable user group remained. However, participants reported that non-usage was not due to dissatisfaction with the system but a reflection of the group’s experience in self-management. It appeared that a major benefit of belonging to the community was simply knowing that there was a resource available if and when it was needed. The small number of participants in the pilot study may also have had a critical effect on the viability and sustainability of this virtual clinic. It is likely that a large and consistent number of active users are required to support a system such as this, particularly to sustain discussion forums.

The discussion forum was the most used, and reported as the most useful, component of the virtual clinic system. This is in agreement with other interventions of this nature [10]. Taking part in peer discussions has shown to help patients with diabetes cope better with their illness [17] and improve adherence to management, resulting in better metabolic control [28]. Participants who answered more questions than they posted found taking part particularly encouraging. A live chat facility was also available to participants but was not used. Users mentioned that they preferred using the asynchronous discussion board as it was easier to follow and they could return to consult the posts in their own time.

Around one-third of the discussion forum posts related directly to issues regarding insulin pumps. These included books on pumping, comfortable sites for positioning the pump, and removing the pump for holidays. By having a site solely for pump users, participants were able to share information and concerns and seek advice from peers in similar situations. Future interventions may consider restricting sites to specific groups, including, for example, newly diagnosed diabetics or teenagers transitioning from pediatric to adult care, so participants can fully benefit from peer support elements.

A number of “lurkers”, people who read but seldom contributed to discussions, were identified in our intervention. Although these users did not actively participate in discussions, many still found them beneficial. Lurking has been described as a form of participation that is both acceptable and beneficial to online groups because information supplied in health-related discussion forums is often used to seek better medical care, and the same information may provide the basis for other discussions in online or offline settings [29]. Furthermore, users in this intervention felt that not contributing offered reassurance in confirming there was nothing else they needed to know.

Participants often felt that involvement in the virtual clinic had not improved their communication with their health care team. Reasons for this included having no need to contact professionals during the intervention and concerns regarding use of the Internet as a means of communication. Those who did use the links suggested they would like to receive more of their routine medical care online. These findings contrast somewhat with studies that suggest enhancing communication with health professionals is a major benefit of Internet-based health care [12]. Participants in the current study had been diagnosed with diabetes for a substantial period of time. It may be that facilities to promote communication with health teams are more useful to those newly diagnosed because their needs are different from someone who has lived with the condition for many years.

The pressure of this intervention on a clinician’s time remains to be assessed. All health care professionals involved in the study were sent questionnaires asking how they spent their time. Generally, these were not returned and highlight the need for effective measures to assess a clinician’s time expenditure. Other studies have found email communication between patients and physicians does not adversely affect a physician’s time [30].

The links to further information did not seem to be used as frequently as the other two components. This may indicate that participants were already well informed about their condition. It has been shown that people with diabetes who are highly educated and have Internet access at home are more likely to search the Web for health information [31].

Users’ responses to the virtual clinic were very positive and provide initial support for the proposal that this intervention can aid self-management. However, there was no evidence in this pilot study of improvements occurring in physiological and psychological measurements. This may be a result of the intervention’s limited timeframe or insufficient usage by participants [32]. It could also be that participants appeared to have had low information needs or that the study excluded those who might benefit more, such as those new to the disease and those new to pumping. Self-efficacy may have declined initially as patients realized that there was more about their condition to learn and understand than previously thought. It is also possible that participants’ increased attention to the topic focused their attention to the fact that diabetes is ultimately an incurable problem. Social and information support was obviously beneficial, but it was not a cure and may have increased participants awareness of all that could “go wrong”.

In addition, the current study’s results revealed high HbA1c measurements with some users disclosing difficulties in managing their diabetes, despite experience of, and adherence to, self-management regimens. Poor metabolic control has been shown to reduce a patient’s quality of life [33] and self-efficacy [34]. In addition, the CIDS scale may not have been the most appropriate instrument to use with insulin pump patients, since some questionnaire content would not apply, such as performing insulin injections.

There were several limitations to this study that would need to be addressed in any future work. There were missing data as we lost four participants to follow up and HbA1c results were only available for eight users post-test. Furthermore, there appeared to be discrepancies between the qualitative and quantitative data findings. The positive tone of the interviews was in contrast to the lack of improvements in self-efficacy and quality of life. This may have been due in part to not having follow-up data for all participants and the small sample size in this pilot study. Future work designed to draw conclusions beyond feasibility and acceptability should incorporate a larger sample. Investigating other relevant outcomes, such as social support, may also be useful in future research.

### Conclusion

Overall this pilot study indicates that a virtual clinic intervention appears to be a feasible and acceptable way to provide patients with the peer support and information necessary to aid self-management. However, no improvements in biological or psychological measures could be confirmed through the data. This may be due to the complex problems this particular group of users faced in achieving good metabolic control, which may subsequently affect their quality of life and self-efficacy. The findings suggest a need for further refinement and testing of the intervention. They are being used to inform further development of Internet-based clinics and the design of larger-scale, controlled intervention studies.

## Abbreviations

ADDQoL: audit of diabetes dependent quality of life
CIDS: confidence in diabetes self-care scale
CSII: continuous subcutaneous insulin infusion
INPUT: promoting insulin therapy
NICE: National Institute of Clinical Excellence
